# Metabolic syndrome and survival of patients with hepatocellular carcinoma: A meta-analysis

**DOI:** 10.3389/fonc.2023.1117846

**Published:** 2023-02-23

**Authors:** Jia Fu, Jinqiong Jiang, Kanghan Liu

**Affiliations:** ^1^ Department of Oncology, Hunan Provincial People’s Hospital (The First Affiliated Hospital of Hunan Normal University), Hunan Normal University, Changsha, China; ^2^ Department of Nephrology and Laboratory of Kidney Disease, Hunan Provincial People’s Hospital (The First Affiliated Hospital of Hunan Normal University), Hunan Normal University, Hunan Clinical Research Center for Chronic Kidney Disease, Changsha, China

**Keywords:** metabolic syndrome, hepatocellular carcinoma, survival, meta-analysis, predictor

## Abstract

**Background:**

Metabolic syndrome (MetS) has been related to a high incidence of hepatocellular carcinoma (HCC). However, the influence of MetS on survival of patients with HCC is still unclear. We performed a systematic review and meta-analysis to evaluate the association between MetS and survival of HCC patients.

**Methods:**

A search of PubMed, Embase, and Web of Science retrieved relevant cohort studies from the inception of the databases to October 16, 2022. Data collection, literature search, and statistical analysis were carried out independently by two authors. We pooled the results using a random-effects model that incorporates heterogeneity.

**Results:**

In the meta-analysis, 8080 patients with HCC were included from ten cohort studies, and 1166 patients (14.4%) had MetS. Eight studies included patients treated primarily with radical hepatectomy, one study with patients receiving sorafenib, and another study included patients who were treated with radical hepatectomy or non-surgical treatments. Pooled results showed that MetS was associated with poor overall survival (OS, risk ratio [RR]: 1.21, 95% confidence interval [CI]:1.08 to 1.37, p = 0.001; I^2^ = 32%) and progression-free survival (PFS, RR: 1.33, 95% CI: 1.18 to 1.49, p < 0.001, I^2^ = 14%). Influencing analysis by excluding one study at a time showed consistent results (p all < 0.05). Subgroup analyses showed similar results in studies with MetS diagnosed with the National Cholesterol Education Program Adult Treatment Panel III or International Diabetes Federal criteria, and in studies with mean follow-up durations < or ≥ 3.5 years (p for subgroup difference all > 0.05).

**Conclusion:**

In patients with HCC, MetS may be a risk factor of poor OS and PFS, particularly for those after radical hepatectomy.

## Introduction

As the sixth most commonly diagnosed cancer among adults and the third leading cause of cancer-related mortality globally, hepatocellular carcinoma (HCC) represents the most common type of liver cancer (1–3). In the current era of HCC treatment, surgical resection, transplantation, interventional chemoembolization, radiofrequency ablation, radiotherapy, and targeted drug therapy are the most common treatments available (4, 5). However, responses of HCC patients to these treatments are varying, and the survival of some patients with HCC remains poor despite of these comprehensive treatments (6, 7). Therefore, uncovering potential risk factors of poor prognosis in patients with HCC is important for risk stratification and development of adjunctive treatments in these patients (8).

Metabolic syndrome (MetS) refers to a cluster of metabolic disorders which involve central obesity, insulin resistance, high blood pressure, and dyslipidemia (9–11). Pathophysiologically, MetS is characterized by insulin resistance and systemic low-degree inflammation, which have been both related to carcinogenesis (12). Epidemiological studies have confirmed the role of MetS as a risk factor for the incidence of various cancers (13, 14), including HCC (15–17). However, for patients with diagnosed HCC, the influence of MetS on their survival remains unclear (18). Some previous studies showed that MetS may be a risk factor of poor survival of patients with HCC (19–23), while other studies did not show similar results (24–28). In this study, we performed a systematic review and meta-analysis to comprehensively investigate the association between MetS and survival of patients with HCC. In addition, the effect of different diagnostic criteria on the association was also explored in subgroup analyses.

## Materials and methods

We followed the instructions of the PRISMA (Preferred Reporting Items for Systematic Reviews and Meta-Analyses) statement (29, 30) and the Cochrane’s Handbook (31) through the meta-analysis. The protocol of the meta-analysis has been registered in the International Platform of Registered Systematic Review and Meta-analysis Protocols (INPLASY, No. 2022120113).

### Literature retrieving

The electronic databases PubMed, Embase, and Web of Science were searched from the inception of the databases until October 16, 2022. A combined search term was used, including (1) “metabolic syndrome” OR “insulin resistance syndrome” OR “syndrome X”; (2) “hepatocellular” OR “liver” OR “hepatic”; (3) “carcinoma” OR “cancer” OR “tumor” OR “malignancy” OR “malignant” OR “neoplasm”; and (4) “survival” OR “death” OR “mortality” OR “prognosis” OR “recurrence” OR “recurrent”. The search was limited to human studies published in full-length articles. No restriction was applied regarding the language of publication. To supplement our search, we manually reviewed the citations of relevant original articles and review articles.

### Study selection

A PICOS-based inclusion criterion was used for this study.

**P (patients)**: Adult patient with confirmed diagnosis of HCC, regardless of the cancer stage or treatments.

**I (exposure)**: Patients with MetS at baseline.

**C (control)**: Patients without MetS at baseline.

**O (outcomes)**: A primary outcome was overall survival (OS), and a secondary outcome was progression-free survival (PFS), compared between HCC patients with and without MetS. Generally, OS was defined as the time elapsed from treatment and to the date of death from any cause, while a PFS is the interval between the initiation of treatment and the first recurrence or progression of the disease.

**S (study design)**: Cohort studies, including prospective and retrospective cohorts;

The diagnosis of MetS was consistent with the criteria used within the included studies. The meta-analysis excluded reviews, preclinical studies, studies involving non-HCC patients, studies lacking the evaluation of MetS, and studies without survival outcomes.

### Data collection and quality assessment

Separately, two authors searched and analyzed literature, collected data, and assessed study quality. A third author was consulted if discrepancies were encountered. Data of study information, patient demographic factors, main treatments, diagnostic criteria of MetS, follow-up durations, outcomes reported, and variables adjusted in the regression model for the analysis of the association between MetS and survival outcomes were collected. An assessment of study quality was done using the Newcastle-Ottawa Scale (32) based on criteria for participant selection, comparability of groups, and validity of results. A study’s quality was determined by the number of stars between 1 and 9, with more stars representing a better study quality.

### Statistical analyses

The main objective was to determine the relative risks of OS and PFS comparing between HCC patients with and without MetS, which were presented as risk ratios (RRs) and the confidence intervals (CIs). By using 95% CIs or p-values, RRs and standard errors (SEs) could be calculated, and a subsequent logarithmical transformation kept the variance stabilized and normalized. Cochrane’s Q test and I^2^ statistics were used to estimate study heterogeneity (33), and the significant heterogeneity is reflected by an I^2^ > 50%. The results were combined using a random-effects model incorporating heterogeneity’s influence (31). A sensitivity analysis that omitted one study at a time was conducted to observe what effect each study has on the overall results (34). Besides, sensitivity analyses were also performed in studies of patients after radical hepatectomy for HCC and in patients who are positive of hepatitis B virus (HBV). In addition, subgroup analyses were conducted to examine how study characteristics influenced the results. Medians of the continuous variables were used as the cutoffs for defining subgroups. An estimate of publication bias was made by constructing funnel plots and applying Egger’s regression asymmetry test to the visual judgment of their symmetry (35). Our analyses were done using RevMan (version 5.1; Cochrane Collaboration, Oxford, UK) and Stata (version 12.0; Stata Corporation, College Station, TX).

## Results

### Studies obtained

**Figure 1:**
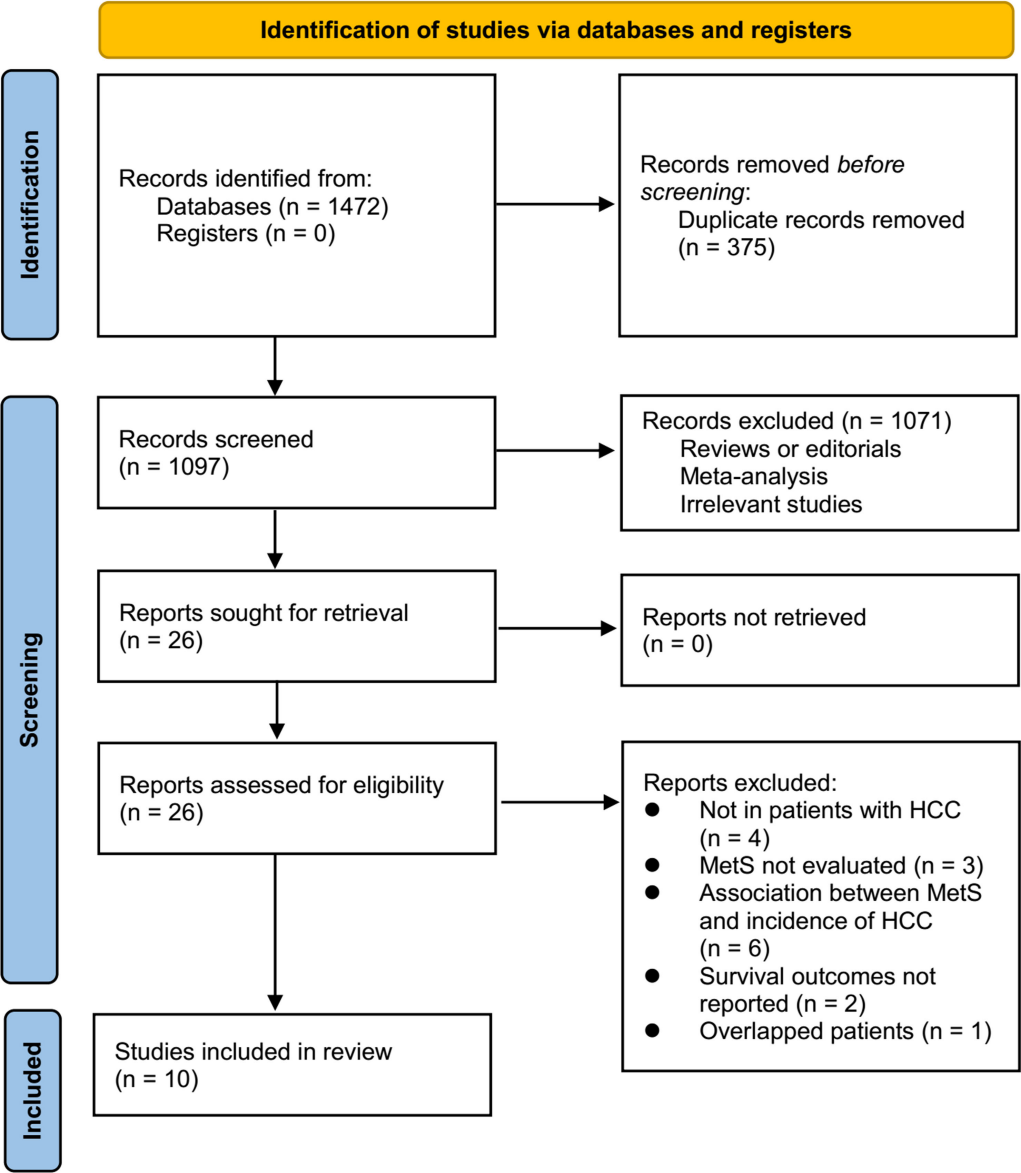
** **A summary of the literature search and study identification process.

**Figure 1** displays the procedure of literature analysis. In short, the initial search of the databases retrieved 1472 articles, and 1097 were left after excluding the duplicated records. In addition, 1071 articles were excluded since their titles and abstracts were not relevant to the meta-analysis, leaving 26 studies in total for the full-text review. Finally, after excluding 16 studies through full-text review, ten studies (19–28) were included. The reasons for the removing of the 16 studies are also presented in **Figure 1**.

### Characteristics of the included studies

As shown in **Table 1**, ten cohort studies, eight retrospective studies (19–25, 27) and two prospective studies (26, 28), involving 8080 patients with HCC were included in the meta-analysis. These studies were published between 2015 and 2022, and performed Italy, Japan, Germany, China, and the United States. All of the studies included patients with HCC, with eight studies with patients treated primarily with surgical resection (19–24, 27, 28), one with sorafenib (25), and another one with surgical resection or non-surgical treatments (26). The sample size of the included studies varied between 56 and 1753. The mean ages of the patients were 50.6 to 70.2 years. The National Cholesterol Education Program Adult Treatment Panel III (NCEP-ATP III) criteria were used for the diagnosis of MetS in seven studies (19, 21–26), while the International Diabetes Federal (IDF) criteria were used in the other three studies (20, 27, 28). Accordingly, 1166 patients (14.4%) had MetS at baseline. The mean follow-up durations varied between 2.2 and 5.0 years. The outcome of OS was reported in all of the included ten studies (19–28), while the outcome of PFS was reported in seven studies (19–24, 27). Multivariate models were applied to analyze the association between MetS and survival outcomes in HCC, and possible confounding factors such as age, sex, hepatic function, tumor size, grade, and treatments etc. were adjusted. The NOS of the included studies were 8 to 9 stars, suggesting moderate to good study quality (**Table 2**).

**Table 1:** Characteristics of the cohort studies enrolled in the meta-analysis.

Study	Country	Design	Patient characteristics	Sample size	Mean age	Men	MetS diagnosis	MetS at baseline	Median follow-up duration	Outcomes reported	Outcome validation	Variables adjusted/matched
Years						%		n (%)	Years				
Vigano 2015 (19)	Italy	RC	Patients received liver resection for HCC	1563	70	79.7	NCEP-ATP III	96 (6.1)	3.7	OS, PFS	Clinical follow-up	Age, sex, MELD score, portal hypertension, stage of fibrosis, major hepatectomy, steatosis, moderate/severe hepatitis, tumor size, multiple nodules, tumor grade, and MVI
Yoshida 2015 (24)	Japan	RC	Patients underwent a curative liver resection for HCC	246	70.2	84.6	NCEP-ATP III	35 (14.2)	3	OS, PFS	Clinical follow-up	Age, sex, BMI, AFP, Child-Pugh Class, major resection, tumor number, size, grade, and MVI
Labenz 2018 (25)	Germany	RC	Patients with advanced HCC treated with sorafenib	152	64.3	87.5	NCEP-ATP III	46 (30.3)	5	OS	Clinical follow-up	Age, sex, PS, Child-Pugh Class, tumor grade, previous treatment and MVI
Tian 2018 (20)	China	RC	Patients undergoing hepatectomy for HCC	1352	50.6	84.2	IDF	198 (14.6)	3.4	OS, PFS	Clinical follow-up	Age, sex, MELD score, cirrhosis, major hepatectomy, tumor size, and MVI
Morisc 2018 (26)	Italy	PC	Patients with HCC undergoing surgical or non-surgical treatments	839	69	78	NCEP-ATP III	295 (35.2)	2.2	OS	Clinical follow-up	Age, sex, MELD score, BCLC class, tumor size, multiple nodules, tumor grade, and metastasis
Tian 2020 (27)	China	RC	Patients undergoing hepatectomy for HCC	746	51.3	83.2	IDF	78 (10.5)	5	OS, PFS	Clinical follow-up	Age, sex, BMI, AFP, Child-Pugh Class, MELD score, ASA class, tumor size and number, and MVI
Rayman 2022 (28)	USA	PC	Patients undergoing robotic hepatectomy for HCC	56	64	64.9	IDF	26 (45.6)	3	OS	Clinical follow-up	Age, sex, BMI, Child-Pugh Class, MELD score, ASA class, previous surgeries, and cirrhosis
Wang 2022 (22)	China	RC	HBV positive patients underwent a curative liver resection for HCC	1753	53	88.9	NCEP-ATP III	163 (9.3)	4.2	OS, PFS	Clinical follow-up	Age, sex, tumor size, Child-Pugh Class, cirrhosis, HBV-DNA, AFP, and vascular invasion
Zhang 2022 (23)	China	RC	HBV positive patients after radical hepatectomy for HCC	389	58.3	87.4	NCEP-ATP III	50 (12.9)	3.4	OS, PFS	Clinical follow-up	Age, sex, smoking, PS, ASA class, Child-Pugh Class, comorbidities, HBV-DNA, antiviral therapy, AFP, portal hypertension, tumor size, multiple nodules, and vascular invasion
Dai 2022 (21)	China	RC	HBV-associated HCC patients after hepatectomy	984	55	76	NCEP-ATP III	179 (18.2)	3.5	OS, PFS	Clinical follow-up	Age, sex, smoking, alcohol drinking, HBV-DNA, antiviral treatment, AFP, tumor size, number of nodule, cirrhosis, Child-Pugh Class, MELD score, surgical procedures, BCLC, and CCI

AFP, alpha-fetoprotein; ASA, American Society of Anesthesiologists; BCLC, Barcelona clinic liver cancer stage; BMI, body mass index; CCI, Charlson Comorbidity Index; HBV, hepatitis B virus; HCC, hepatocellular carcinoma; IDF, International Diabetes Federal; MetS, metabolic syndrome; MELD, Model for End-stage Liver Disease; MVI, microvascular invasion; NCEP-ATP III, National Cholesterol Education Program Adult Treatment Panel III; OS, overall survival; PC, prospective cohort; PFS, progression-free survival; PS, performance status; RC, retrospective cohort.

**Table 2:** Study quality evaluation *via* the Newcastle-Ottawa Scale.

Study	Representativeness of the exposed cohort (a)	Selection of the non-exposed cohort (b)	Ascertainment of exposure (c)	Outcome not present at baseline (d)	Control for age and sex (e)	Control for other confounding factors (f)	Assessment of outcome (g)	Enough long follow-up duration (h)	Adequacy of follow-up of cohorts (i)	Total
Vigano 2015 (19)	1	1	1	1	1	1	1	1	1	9
Yoshida 2015 (24)	1	1	1	1	1	1	1	1	1	9
Labenz 2018 (25)	0	1	1	1	1	1	1	1	1	8
Tian 2018 (20)	1	1	1	1	1	1	1	1	1	9
Morisc 2018 (26)	1	1	1	1	1	1	1	0	1	8
Tian 2020 (27)	1	1	1	1	1	1	1	1	1	9
Rayman 2022 (28)	0	1	1	1	1	1	1	1	1	8
Wang 2022 (22)	1	1	1	1	1	1	1	1	1	9
Zhang 2022 (23)	1	1	1	1	1	1	1	1	1	9
Dai 2022 (21)	1	1	1	1	1	1	1	1	1	9

Definition of items of the Newcastle-Ottawa Scale in this meta-analysis: a, one star if patients were consecutively included or randomly selected; b, one star if the non-exposed cohort was drawn from the same community as the exposed cohort; c, one star if the exposure was based on secure record or structured interview; d, one star because survival outcomes were observed; e, one star if age and sex were adjusted; f, one star if other tumor variables were adjusted (stage, grade, tumor size, number of nodule, etc.); g, one star if the outcome was independently or blindly assessed, or evidenced via record linkage; h, one star if the median follow-up duration was at least one year; i, one star if complete follow up- all subject accounted for, or the number of lost less than or equal to 20% or description of those lost suggested no different from those followed.

### MetS and OS of patients with HCC

**Figure 2:**
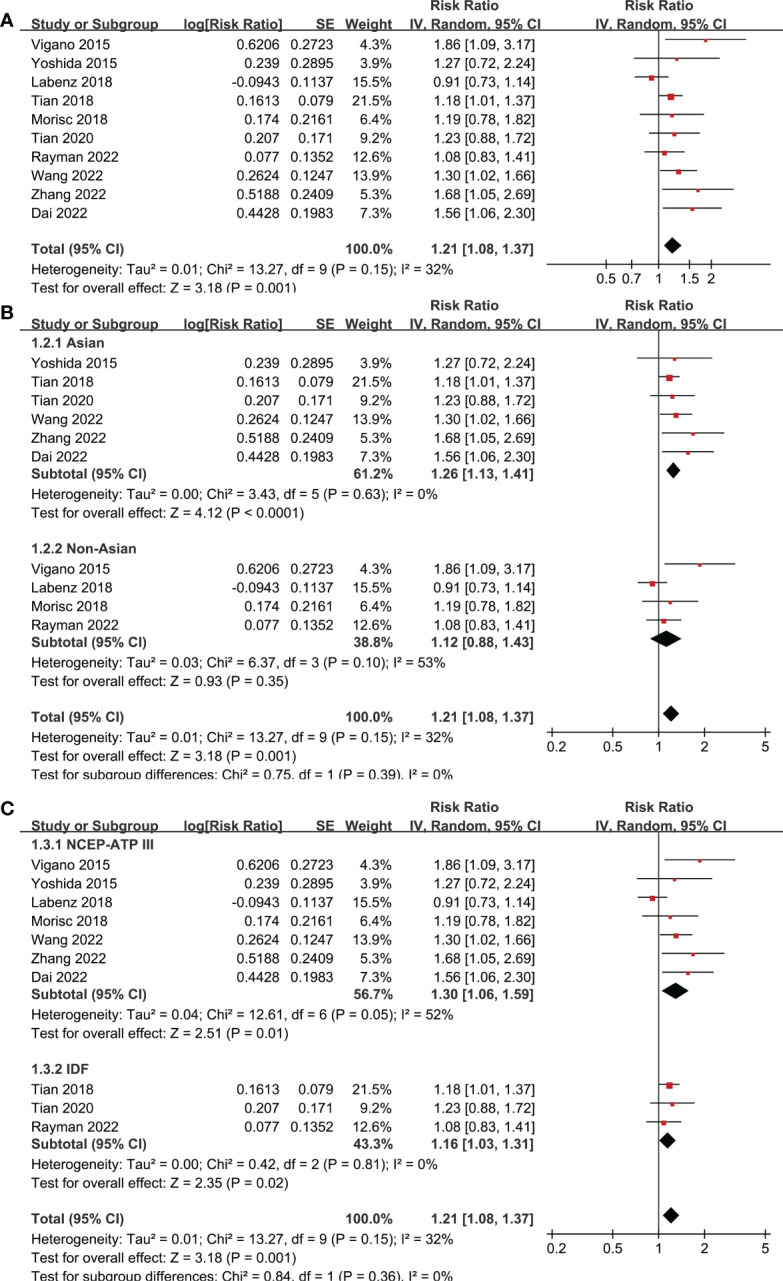
Forest plots for the meta-analyses regarding the association between MetS and OS in patients with HCC. **(A)** overall meta-analysis; **(B)** subgroup analysis according to the study country; and **(C)** subgroup analysis according to the diagnostic criteria for MetS.

**Figure 3:**
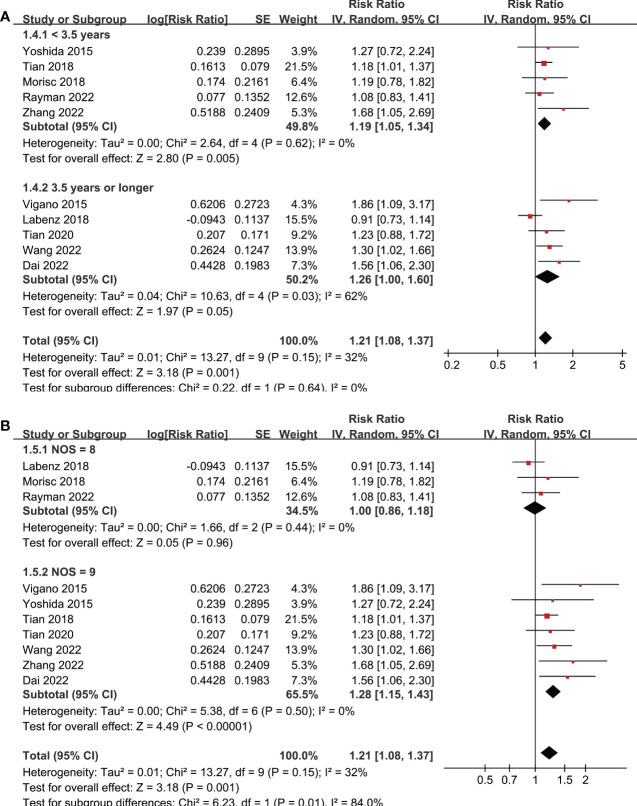
Forest plots for the subgroup-analyses regarding the association between MetS and OS in patients with HCC. **(A)** subgroup analysis according to the follow-up durations; and **(B)** subgroup analysis according to the study quality scores.

Ten studies reported the association between MetS and OS in patients with HCC (19–28). Pooled results showed that MetS was associated with poor OS of HCC (RR: 1.21, 95% CI: 1.08 to 1.37, p = 0.001; **Figure 2A**) with mild heterogeneity (p for Cochrane’s Q test = 0.15; I^2^ = 32%). Sensitivity analysis by excluding one study at a time showed consistent results (RR: 1.18 to 1.25, p all < 0.05). Sensitivity limited to the eight studies (19–24, 27, 28) only included patients after hepatectomy for HCC showed similar results (RR: 1.25, 95% CI: 1.13 to 1.38, p < 0.001; I^2^ = 0%). Similarly, sensitivity analyses limited to the three studies (21–23) including only HBV-positive patients also showed similar results (RR: 1.41, 95% CI: 1.17 to 1.71, p < 0.001; I^2^ = 0%). Subgroup analyses according to study country (**Figure 2B**), diagnostic criteria of MetS (**Figure 2C**), and follow-up durations (**Figure 3A**) showed similar results (p for subgroup difference all > 0.05). However, subgroup analysis according to study quality showed that MetS was associated with poor OS in studies of 9 points in NOS (RR: 1.28, 95% CI: 1.15 to 1.43, p < 0.001; I^2^ = 0%), but not in studies of 8 points in NOS (RR: 1.00, 95% CI: 0.86 to 1.18, p = 0.96; I^2^ = 0%; p for subgroup difference = 0.01; **Figure 3B**).

### MetS and PFS of patients with HCC

**Figure 4:**
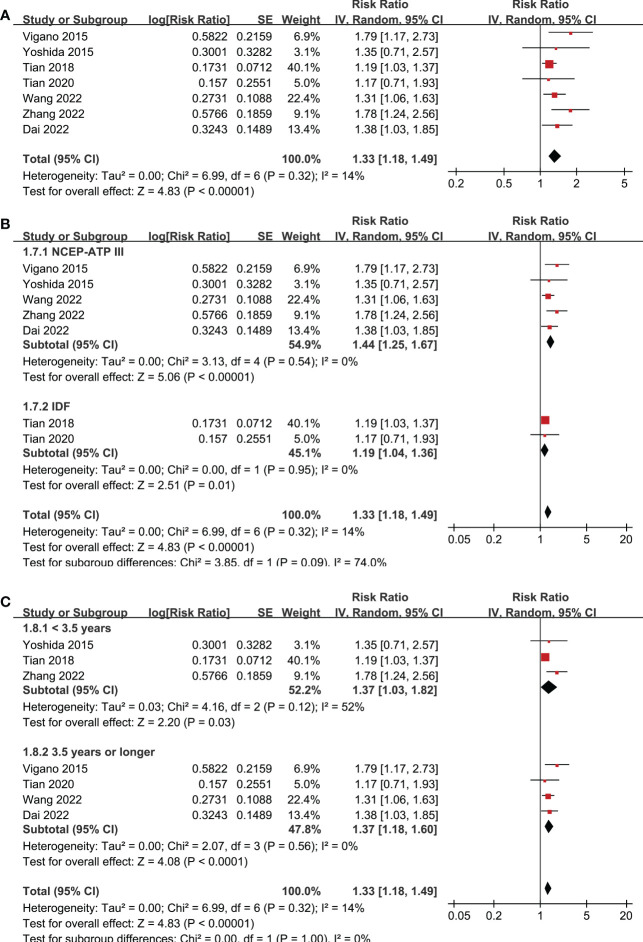
Forest plots for the meta-analyses regarding the association between MetS and PFS in patients with HCC. **(A)** overall meta-analysis; **(B)** subgroup analysis according to the diagnostic criteria for MetS; and **(C)** subgroup analysis according to the follow-up durations.

The outcome of PFS was reported in seven studies (19–24, 27), which all included patients after hepatectomy for HCC. Pooled results of seven studies, all with NOS of 9 points, showed that MetS was associated with poor PFS (RR: 1.33, 95% CI: 1.18 to 1.49, p < 0.001, I^2^ = 14%; **Figure 4A**) in patients with HCC. Sensitivity analysis by omitting one study at a time also showed consistent results (RR: 1.27 to 1.42, p all < 0.05). Sensitivity analyses limited to the three studies (21–23) including only HBV-positive patients also showed similar results (RR: 1.41, 95% CI: 1.21 to 1.65, p < 0.001; I^2^ = 0%). Subgroup analyses according to the diagnostic criteria of MetS and follow-up durations showed similar results (p for subgroup difference all > 0.05; **Figures 4B, C**).

### Publication bias

**Figure 5:**
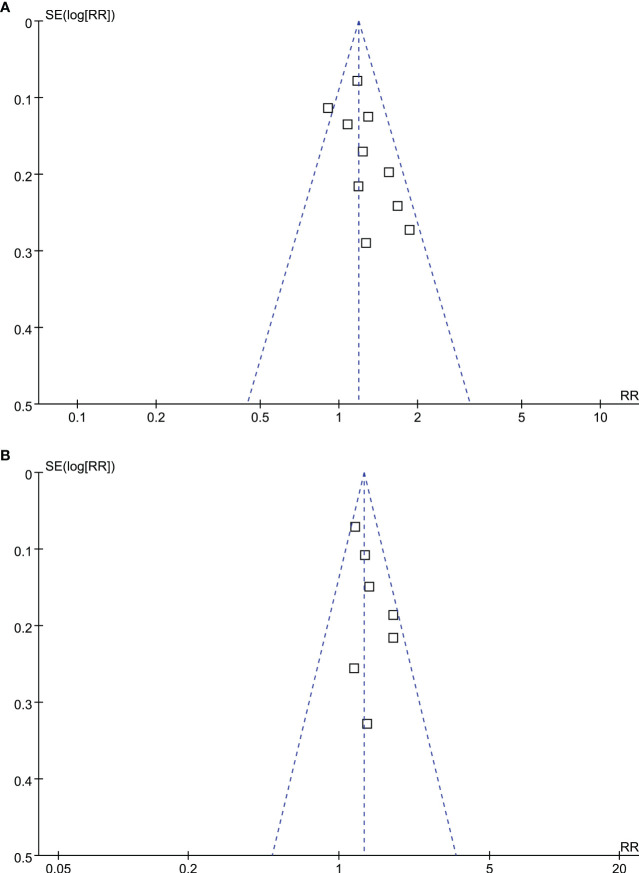
Funnel plots for the publication bias underlying the meta-analyses; **(A)** funnel plots for the meta-analysis of OS; and **(B)** funnel plots for the meta-analysis of PFS.

According to **Figures 5A, B**, funnel plots for OS and PFS outcomes show symmetry, indicating low risks of publication bias. Results of Egger’s regression tests also indicated low risks of publication bias underlying the meta-analyses for the associations between MetS with OS and PFS of HCC patients (p = 0.29 and 0.37, respectively).

## Discussion

Researches regarding the roles of metabolic disorders in the pathogenesis and progression of cancer have become a hotspot in recent decades. As a syndrome integrating multiple common metabolic disorders, MetS has been shown to be closely related to the incidence of multiple cancers (36–40), but not all (41). Besides, in view of the high prevalence of MetS in patients with confirmed diagnosis of cancer (42), it is also important to determine the influence of MetS on survival of these patients. Previous studies have shown that MetS may be a predictor of poor survival in patients with breast cancer (43), prostate cancer (44), colorectal cancer (45), and gastric cancer (46). In this meta-analysis, by combining the results of ten available cohort studies, we further confirmed that MetS was also independently associated with poor OS and PFS in patients with HCC, particularly in patients after radical hepatectomy for HCC. These findings further expanded the role of MetS in HCC, which suggested that besides of being a risk factor of high incidence of HCC (15, 16) MetS may also be a predictor of poor survival of patients with confirmed diagnosis of HCC.

To the best of our knowledge, this study may be the first meta-analysis which evaluated the association between MetS and survival in patients with HCC. The strengths of the meta-analysis included the followings. First, extensive literature search was performed in three electronic databases, which retrieved ten up-to-date studies relevant to the aim of the meta-analysis. Second, all of the included studies were cohort studies, which could provide a longitudinal relationship between MetS and poor survival of patients with HCC. Third, multivariate regression analyses were used in all of the included studies when the association between MetS and survival outcome of patients with HCC was investigated. After adjustment of potential confounding factors such as age, sex, tumor characteristics, and therapy, results of the meta-analysis may suggest an independent association between MetS and poor survival in patients with HCC. Finally, consistent results were obtained in sensitivity analyses by excluding one study at a time and in subgroup analyses according to predefined study characteristics such as definition of MetS and follow-up durations. These findings may indicate the robustness of the meta-analysis results. Interestingly, subgroup analysis according to study quality showed that MetS was associated with poor OS in studies of 9 points in NOS, but not in studies of 8 points in NOS. These findings may further reflect the reliability of the meta-analysis results regarding OS, which were supported by high-quality studies. For the three studies with 8 points of quality score (21–23), they were either exposed to patient selection bias or short follow-up duration, which may lead to bias of the results.

The mechanisms underlying the association between MetS and poor survival of patients with MetS may be multifactorial. Pathophysiologically, MetS is characterized by low-grade systemic inflammation and insulin resistance, both of which may adversely affect the progress of HCC. For example, MetS has been related to a variety of inflammatory signaling pathways, such as tumor necrosis factor alpha, interleukin-1, toll-like receptors, C-type lectin receptors, and nuclear factor kappa-light-chain-enhancer of activated B cells etc., all of which have been documented to be involved in the development of the malignant properties of HCC, including promoting proliferative and survival signaling, inducing angiogenesis, evading immune surveillance, activating invasion and metastasis, and inducing genome instability (47, 48). Insulin resistance has been related to the generation of multidrug resistance of HCC in some experimental studies (49). In addition, hyperinsulinemia and subsequent insulin resistance has been revealed as a promoting factor for maintaining the homeostasis in the endoplasmic reticulum by regulating autophagy, enhancing the survival of HCC (50). Moreover, a recent study showed that insulin resistance as indicated by a higher level of Homeostatic Model Assessment of Insulin Resistance (HOMA-IR), was a significant predictor for HCC recurrence after curative treatment (51). Finally, some components of MetS have also been suggested to adversely affect the survival of patients with HCC, including visceral adiposity (52), diabetes (53), and dyslipidemia (54), which may also partly explain the association between MetS and poor survival in patients with HCC.

Results of our meta-analysis also have limitations. Firstly, eight of the ten included studies were retrospective cohort studies, which may be associated with the risks of selection and recall biases. Accordingly, results of the meta-analysis should be validated in large-scale prospective studies. Secondly, because all of the included studies focused on the tumor-related outcome of the patients, and the results of the meta-analysis showed that MetS was associated with both poor OS and PFS in patients with HCC, it could be concluded that tumor progression and related death was likely to increase in HCC patients with MetS. It could not be determined at current stage whether non-cancer related death (such as cardiovascular death) was also increased in HCC patients with MetS. Further studies are needed in this regard. Thirdly, eight of the studies included HCC patients after surgical resection. We could not determine whether the association between MetS and prognosis of HCC was consistent in HCC patients who received other non-surgical treatments, such as transarterial chemoembolization. Large-scale prospective studies are needed to determine the influences of different treatment strategies on the association between MetS and survival of patients with HCC. To the best of our knowledge, no study to date has observed the potential influence of MetS on survival of liver transplantation (LT) solely in HCC patients. A recent study (55) showed that for patients receiving LT (47% with HCC), MetS at baseline is associated with a higher risk of post-LT morbidity yet without affecting mortality. Large prospective studies are also needed to determine if MetS may affect the OS and PFS in HCC patients after LT, which may interesting in the debate of LT versus hepatectomy for HCC on MetS. Moreover, the etiologies of HCC are multifactorial, and the different etiologies to HCC may affect the association between MetS and prognosis of HCC. Although our sensitivity analyses showed consistent association between MetS and poor survival of HCC in HBV-positive patients, it remains unclear whether the association was similar for patients with other risk factors/etiologies for HCC, such as those with HCV-positivity, alcoholic liver disease, nonalcoholic fatty liver disease, aflflatoxin exposure, hemochromatosis, and others (56). Studies are needed in the future for further investigation. In addition, although results of the meta-analysis were obtained on the basis of studies with multivariate analyses, we could not exclude the residual confounding factors that may affect the association between MetS and survival of HCC patients, such as the concurrent medications. For example, satin use has been related to reduced mortality and recurrence of HCC (57). Finally, as a meta-analysis of observational studies, we could not determine if the association between MetS and poor survival of HCC is causative. Clinical studies may be considered to investigate whether interventions targeting the metabolic disorders in MetS could favorably influence the clinical outcome of patients with HCC.

In conclusion, results of the meta-analysis indicate that MetS may be independently associated with poor OS and PFS in patients with HCC, particularly for patients after radical hepatectomy for HCC. Future studies are needed to determine the underlying mechanisms, and to investigate the potential benefits of interventions targeting the metabolic disorders in patients with HCC.

## Author contributions

JF and KL designed the study. JF and JJ performed database search, literature review, data collection, and study quality evaluation. JF and KL performed statistical analyses and interpreted the results. JF drafted the manuscript. All authors revised the manuscript and approved the submission.
